# Arbitrary Multicolor Photodetection by Hetero-integrated Semiconductor Nanostructures

**DOI:** 10.1038/srep02368

**Published:** 2013-08-06

**Authors:** Liwen Sang, Junqing Hu, Rujia Zou, Yasuo Koide, Meiyong Liao

**Affiliations:** 1 State Key Laboratory for Modification of Chemical Fibers and Polymer Materials, College of Materials Science and Engineering, Donghua University, Shanghai 201620, China; 2 National Institute for Material Science (NIMS), 1–1 Namiki, Tsukuba, Ibaraki, 305-0044, Japan

## Abstract

The typical photodetectors can only detect one specific optical spectral band, such as InGaAs and graphene-PbS quantum dots for near-infrared (NIR) light detection, CdS and Si for visible light detection, and ZnO and III-nitrides for UV light detection. So far, none of the developed photodetector can achieve the multicolor detection with arbitrary spectral selectivity, high sensitivity, high speed, high signal-to-noise ratio, high stability, and simplicity (called 6S requirements). Here, we propose a universal strategy to develop multicolor photodetectors with arbitrary spectral selectivity by integrating various semiconductor nanostructures on a wide-bandgap semiconductor or an insulator substrate. Because the photoresponse of each spectral band is determined by each semiconductor nanostructure or the semiconductor substrate, multicolor detection satisfying *6S* requirements can be readily satisfied by selecting the right semiconductors.

Multicolor optical sensing with high sensitivity at designed wavelengths can be applied in a variety of applications, such as imaging, surveillance, optical communication, remote control, and target identification^1^,^2^,^3^,^4^. However, the typical photodetectors can only detect one specific optical spectral band, such as InGaAs and graphene-PbS quantum dots for near-infrared (NIR) light detection, CdS and Si for visible light detection, and ZnO and III-nitrides for UV light detection^5^,^6^,^7^,^8^,^9^,^10^. Generally speaking, an ideal photodetector should have spectral *selectivity*, high *sensitivity*, high *speed*, high *signal-to-noise ratio*, high *stability (i.e. high temperature)*, and *simplicity for fabrication* (called *6S* requirements)^11^. However, none of the developed photodetector can achieve the multicolor detection with arbitrary spectral selectivity while maintaining other high photoresponse performance. Several routes have been proposed to perform dual- or multicolor detection at specific wavelengths. One conventional approach is to utilize multiple stacks of optically absorbing materials in a thin-film structure, such as a quantum-well photodetector using interband/intraband transitions or homo-/heterojunction structures^12^,^13^,^14^. This stacking approach relies on the quality of the interface between each absorption layer, which makes fabrication difficult and complex, especially for large lattice-mismatched heterojunctions. This restricts photodetectors that respond over a wide absorption range. Another method is to dope semiconductors so that the cut-off wavelength can be shifted to longer wavelengths. However, this method induces gap states that degrade device performance, such as the response speed. Though an alternative organic material has been proposed to achieve full-spectrum photodetection from NIR to UV^15^, organic materials are notoriously thermally unstable. In particular, the dark current from such an organic material is quite high. Another strategy is structural modification of a certain material, such as graphene to achieve multiband detection^16^. However, wide-range control over an energy bandgap without inducing fatal defects is impossible to achieve using this method. Compositionally tunable semiconductor nanostructures provide a promising route for multicolor photodetection^17^,^18^,^19^. For example, compositionally graded CdS_x_Se_1-x_ nanowire parallel arrays were fabricated for photodetection by Takahashi *et al*.^19^ However, the reported CdS_x_Se_x-1_ was utilized for a specific optical band photodetection in the visible light region. It is difficult to extend the optical band to deep-UV (DUV) or IR region by using CdS_x_Se_1-x_ only. Yang's group tuned the bandgap of In_x_GaN_1-x_ nanowires from infrared to UV region by changing the In content^18^. It is a pity that, no further efforts have been made for multicolor photodetection through hybrid integration by using In_x_Ga_1-x_N. Therefore, it has been a significant challenge to perform multicolor photodetection with wide spectral range (i,e, DUV to IR) that satisfies the *6S* requirements.

In this work, we report a universal strategy for multicolor photodetection with arbitrary spectral selectivity by integrating various semiconductor nanostructures on a wide-bandgap semiconductor or an insulator substrate. Each semiconductor nanostructure or the substrate governs the photoresponse properties without influencing each other due to the small area of the nanostructure in the hetero-integrated photodetector. By properly selecting the semiconductors with different bandgaps and high crystal quality, multicolor detection can be achieved with satisfying the 6S requirements.

## Results

**Figure 1:**
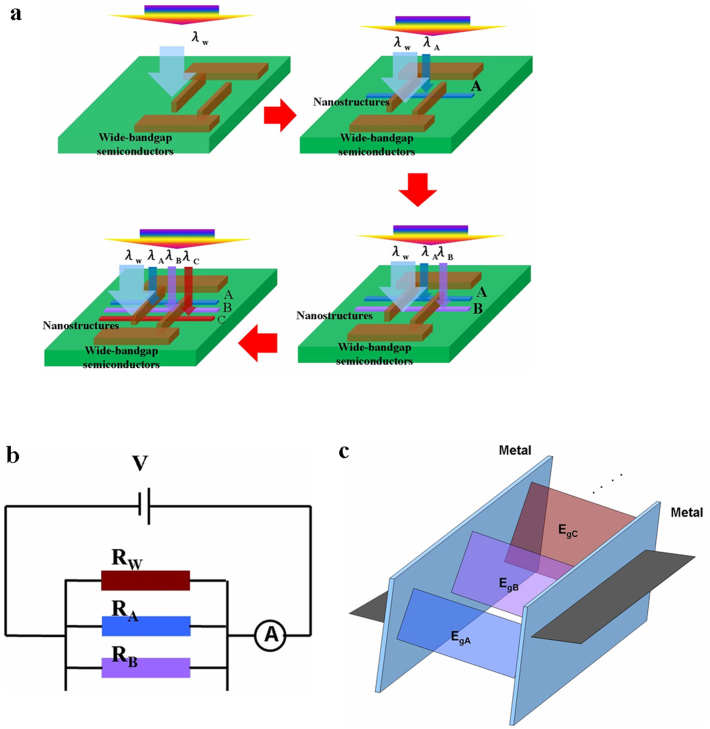
Schematic concept of the photodetectors for one-band, two-band, and multiband detection. (a) Single-band ultraviolet (UV) or deep-ultraviolet (DUV) detection is performed by the wide-bandgap semiconductor substrate; two-band detection is performed by placing a nanostructured semiconductor with a narrower bandgap on the wide-bandgap semiconductor; multiband detection is achieved by placing two or more nanostructured semiconductors on the wide-bandgap semiconductor. (b) Different semiconductors nanostructures response to the optical light separately without charge transfer. (c) The energy band diagram reveals that the Fermi level of each semiconductor is determined by each semiconductor and the metal.

In our strategy, various nanostructured semiconductors with different bandgaps are simultaneously integrated into the same insulating substrate with a two-terminal device structure for different spectral selectivities. The key features of this approach are as follows: (i) each semiconductor is only sensitive to a specific spectral range and does not affect any other semiconductor; (ii) the interface states between two different semiconductor nanostructures can be ignored; and (iii) each semiconductor nanostructure exhibits high crystal quality, ensuring high-performance photodetection over a specific spectral band. The methods of hetero-integrating nanostructured semiconductors on a wide-bandgap semiconductor (NOW) substrate or on another semiconductor substrate (SOS) are developed here. The schematic device configuration is illustrated in Fig. 1. Our approach is clearly different from approaches reported for semiconductor nanostructure-based photodetectors, which normally integrate one nanostructure on an insulating substrate and can only detect a specific spectral band^20^,^21^,^22^. Wide-bandgap semiconductors such as diamond or III-nitrides can not only act as substrates but also as UV-detecting materials, owing to their low dark current and high responsivity in UV detection. In our study, various one-dimensional or quasi-one-dimensional nanostructures with different energy bandgaps, such as CdS (E_g _ = 2.4 eV) nanobelts, SnO_2_ (E_g _ = 3.3 eV) nanowires, ZnO (Eg = 3.4 eV) tetrapod-branched sub-microrods, and Ga_2_O_3_ (E_g_ = 4.9 eV) nanobelts, were utilized to demonstrate multicolor photodetection. Numerous studies have indicated that even above the quantum confinement size regime, nanostructured photodetectors can yield a higher photosensitivity than their bulk counterparts due to their large surface-to-volume ratio and nearly defect-free single-crystal characteristics^23^,^24^,^25^,^26^,^27^,^28^.

### Extension of the UV range detection

**Figure 2:**
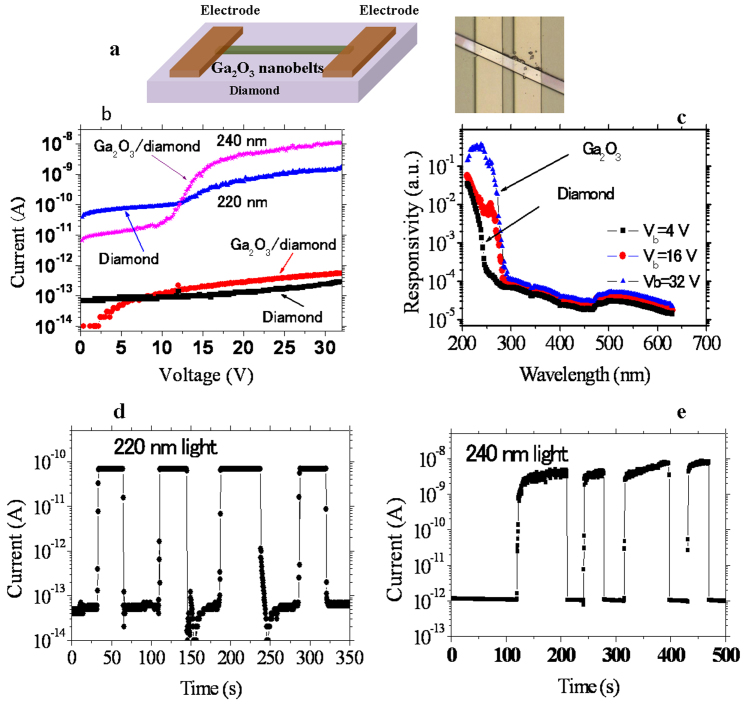
DUV and UV two-band photodetectors created by integrating Ga_2_O_3_ nanobelts on an intrinsic single-crystal diamond layer. (a) Schematic showing and optical image of the device structure. (b) Dark current and photocurrent dependence on the applied voltage characteristics of the Ga_2_O_3_/diamond hetero-integrated photodetector and diamond photodetector, respectively. The devices are illuminated with either DUV or UV light. (c) Spectral response of the two-band photodetectors at various voltages, demonstrating that the spectral response can be tailored by changing the applied voltage. (d) and (e) Time response of the device under 220 nm (V_b_ = 4 V) and 240 nm (V_b_ = 32 V) light illumination, respectively, demonstrating that the fast photoresponse is retained.

By integrating a wide-bandgap semiconductor nanostructure on a super-wide-bandgap semiconductor as a photodetector, the UV range can be broadened. Single-crystal diamond, with a wide bandgap of 5.5 eV, offers the highest figure of merit for high-performance solar-blind deep-UV (DUV) photodetectors meeting the *6S* requirement^29^,^30^. Monoclinic gallium oxide (*β*-Ga_2_O_3_), with a bandgap of 4.2–4.9 eV, also exhibits high intrinsic resistance and high spectrum selectivity. *β*-Ga_2_O_3_ nanobelts were prepared by a high-temperature thermal reaction inside an alumina tube (Supplementary Fig. S1). By integrating a *β*-Ga_2_O_3_ nanobelt on an intrinsic diamond epilayer, dual-wavelength solar-blind photodetectors with a cut-off wavelength of around 280 nm were demonstrated. The device consists of a single-crystal *β*-Ga_2_O_3_ nanobelt placed on a type-IIa single-crystal diamond film, as shown in Fig. 2a. Due to the small volume of the nanostructure, the optical absorption of diamond will rarely be affected.

The hetero-integrated *β*-Ga_2_O_3_/diamond device showed an extremely low dark current (0.55 pA at a voltage of V_b_ = 32 V), as shown in Fig. 2b. Therefore, a high photocurrent-to-dark current ratio greater than 10^3^ and 10^4^ at V_b_ = 32 V was achieved when the device was exposed to 220- (diamond band edge) and 240-nm (*β*-Ga_2_O_3_ band edge) light illumination, respectively, as shown in Fig. 2b. The quantum efficiency of diamond under 220-nm light irradiation (~10 μW/cm^2^) reached 10^3^% and that at 240 nm (30 μW/cm^2^) for the *β*-Ga_2_O_3_ nanobelt was as high as 10^6^%. By using the difference in the interfaces of the metal/diamond structure and metal/Ga_2_O_3_ nanobelt, the photoresponse spectra could be tailored by the applied voltage (Fig. 2c). It is noted that the spectral response was calibrated by the incident photon flux of each wavelength. At relatively low voltages (e.g., 4 V), the dominating cut-off wavelength of the *β*-Ga_2_O_3_/diamond photodetector is located at 225 nm, which is attributed to absorption from diamond. At longer wavelengths, the photoresponse of the *β*-Ga_2_O_3_ nanobelt is activated and becomes stronger as the applied voltage increases. At 16 V, the responses at 220 nm and 240 nm are comparable. Further increasing the voltage to 32 V renders the response from the *β*-Ga_2_O_3_ nanobelt dominant. This property was demonstrated by the current-voltage (I–V) characteristics upon exposure to the 220 and 240 nm light illumination, as shown in Fig. 2b, in which the I–V characteristics at 220 nm are saturated at higher voltages. The I–V characteristics under light illumination are similar to those of each semiconductor alone. This suggests that no additional defects are induced between the *β*-Ga_2_O_3_ nanobelt and diamond. It should be noted that, to normalize the spectral response, the response over the entire surface of the electrode on diamond was calculated. For the *β*-Ga_2_O_3 _nanobelts, the effective absorption area is much smaller. Therefore, the real responsivity of the *β*-Ga_2_O_3_ nanobelts is much higher than that shown in Fig. 2c. The discrimination ratio between DUV and visible light reaches four orders of magnitude, which satisfying the DUV detection under visible-blind condition.

The hetero-integration process does not affect the fast response speed of the *β*-Ga_2_O_3_/diamond detector. Fig. 2d and E plots the time response at bias voltages of 4 V and 32 V upon 220 nm and 240 nm light illumination, respectively. As seen, both of the electrical currents drop to nearly their original values within 0.3 s (limitation of the measurement system) once the light is mechanically turned off. Such a fast response is determined by each given material (Supplementary Fig. S5 and S7). All of these results indicate that the photoresponse properties of each semiconductor in the *β*-Ga_2_O_3_/diamond dual-wavelength photodetector can be maintained by using the current strategy. The extension of the response in the UV band benefits to the visible-blind detection, i.e., flame detection, where visible light response should be avoided.

### Visible and deep-UV dual-band photodetection

**Figure 3:**
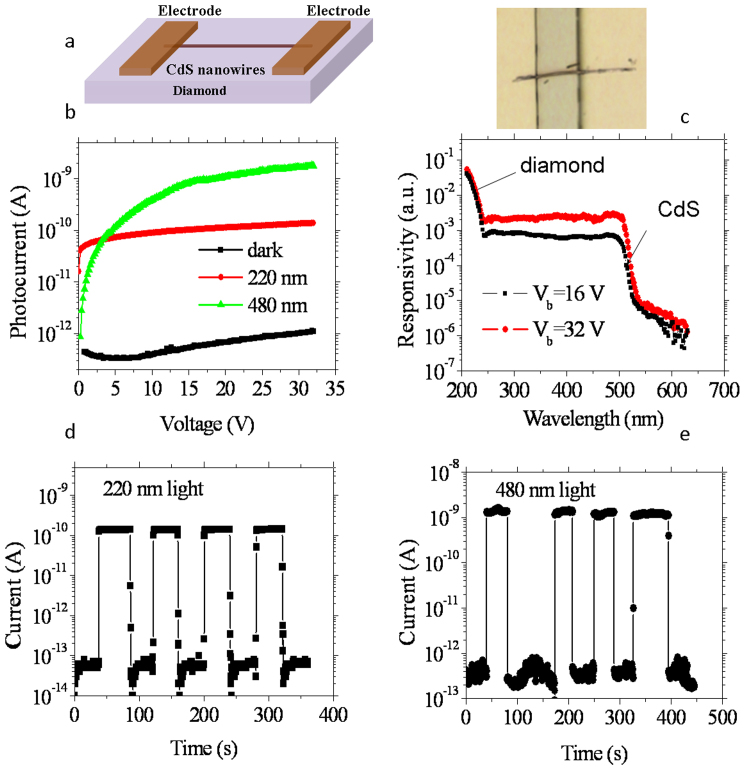
Visible light and DUV light two-band photodetectors created by integrating CdS nanowires on an intrinsic single-crystal diamond layer. (a) Schematic showing and optical image of the device structure. (b) Dark current and photocurrent dependence on the applied voltage characteristics of the CdS/diamond hetero-integrated photodetector. The device was illuminated with either DUV or visible light. (c) Spectral response of the two-band photodetectors at 16 and 32 V, demonstrating the two-band response to visible and DUV light. (d) and (e) Time response of the device under 220 nm (V_b_ = 32 V) and 480 nm (V_b_ = 32 V) light illumination, respectively, demonstrating that the fast photoresponse is retained.

High-performance dual-band photodetectors for visible light and DUV light detection can also be developed by integrating a narrow-bandgap nanostructured semiconductor on a diamond intrinsic layer. For example, the II–VI semiconductor CdS, with a direct bandgap of 2.5 eV, has been utilized to this end; the detector showed a high spectral responsivity (quantum efficiency > 10^7^ at 490 nm: 1.5 mW/cm^2^) in the blue-green region^31^. CdS nanowires were synthesized through a simple hydrothermal reaction (Supplementary Fig. S3). The integrated CdS/diamond device is shown in Fig. 3a. The dark current of the CdS/diamond hetero-integration photodetector slightly increased to 1.1 pA at 32 V relative to the current recorded for the diamond photodetector itself (Fig. 3b), which originated from the CdS nanowire (Supplementary Fig. S8). The spectral responses of the CdS/diamond photodetector at bias voltages of 16 V and 32 V are shown in Fig. 3c. The two-band response to visible light and DUV light of CdS and diamond, respectively, are clearly shown. The responsivities to 220 and 480 nm light are dependent on the applied voltage. Upon illumination with 220 nm light, the photocurrent from the diamond film shows saturated I–V characteristics at higher voltages, while the photocurrent from the CdS nanowires under 480-nm light illumination clearly increases further at higher voltages (Fig. 3a). As a result, at 32 V, the discrimination ratio between 210/400 nm is around 14; while at 16 V, this discrimination ratio increases to 57. We mention that the spectral response at longer wavelength (> 250 nm) was largely underestimated. This is because the diamond device area rather than that of the individual nanowire was utilized. If hundreds of or more CdS nanowires are integrated, the long wavelength response would be enhanced even though the diamond device area is adopted for practical applications. The response speeds for the absorption of the two wavelengths are also maintained, as shown in Fig. 3d and e. All of these properties of the CdS/diamond detectors unambiguously confirm again that the characteristics of dual-wavelength photodetectors only rely on the absorption of each semiconductor material.

**Figure 4:**
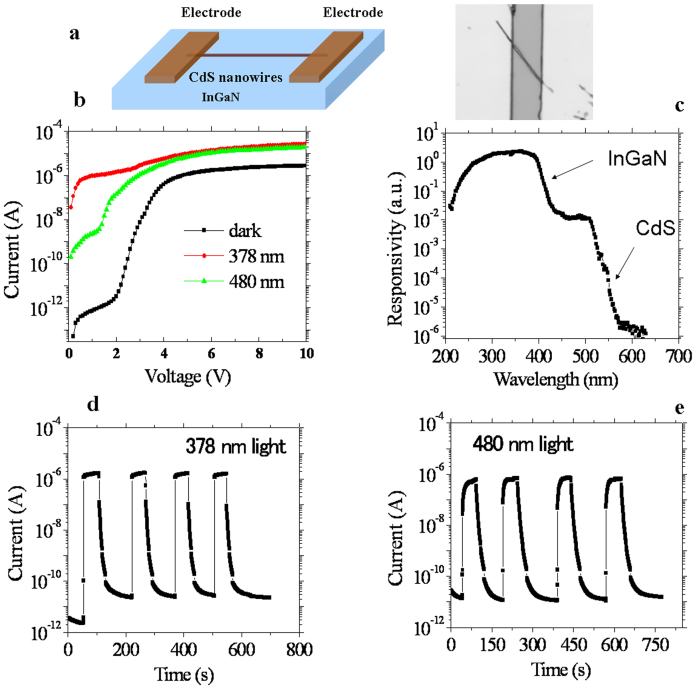
Visible light and UV light two-band photodetectors created by integrating CdS nanowires on an InGaN layer. (a) Schematic showing and optical image the device structure. (b) Dark current and photocurrent dependence on the applied voltage characteristics of the CdS/InGaN hetero-integrated photodetector. The devices were illuminated with either UV or visible light. (c) Spectral response of the two-band photodetectors, demonstrating the two-band response to visible and UV light. (d) and (e) Time response of the device under 378 nm (V_b_ = 2 V) and 480 nm (V_b_ = 2 V) light illumination, respectively, demonstrating that the transient response properties of each semiconductor are retained.

The spectral range of hetero-integration photodetectors can also be extended by varying the substrate material. In_x_Ga_1-x_N compounds with tunable direct bandgaps are promising in the detection of UV light from 320 nm to longer wavelengths^32^. By integrating CdS nanowires on a high-quality InGaN film, dual-band photodetectors sensitive to light from the UV-A to the visible light region can be achieved. An as-fabricated hetero-integrated CdS/InGaN photodetector is demonstrated in Fig. 4a. The CdS nanowire does not affect the diamond photocurrent-voltage characteristics upon DUV light illumination, as shown in Fig. 4b. Fig. 4c shows the photoresponse spectrum of the CdS/InGaN hetero-integrated photodetector at a bias voltage of 2 V, clearly exhibiting a dual-band response from either the InGaN film in the UV-A region or the CdS nanowires in the blue-green region. The quantum efficiency (2000%@378 nm: ~0.5 mW/cm^2^) of the InGaN is also maintained. Although InGaN-based photodetectors suffer from a large leakage current due to the nature of InGaN films, the ratio of the photocurrent to dark current at 2 V is greater than 10^4^ at 480 nm (absorption from CdS nanowires) and 10^5^ at 378 nm (absorption from InGaN film). The temporal responses to shutting on/off the light at wavelengths of 378 nm and 480 nm at a bias voltage of 2 V are shown in Fig. 4c and d, which are also governed by each individual semiconductor (Supplementary Fig. S6 and S8). The slow components of the current drop observed after shutting off the illumination are due to the existence of traps in the InGaN films^33^. We note that the area of the nanostructures is much smaller than that of the substrate covered by the electrode. Even though the substrate area is utilized for the calibration of the spectral response, multicolor photodetection can be still clearly distinguished.

### Three-band photodetection

**Figure 5:**
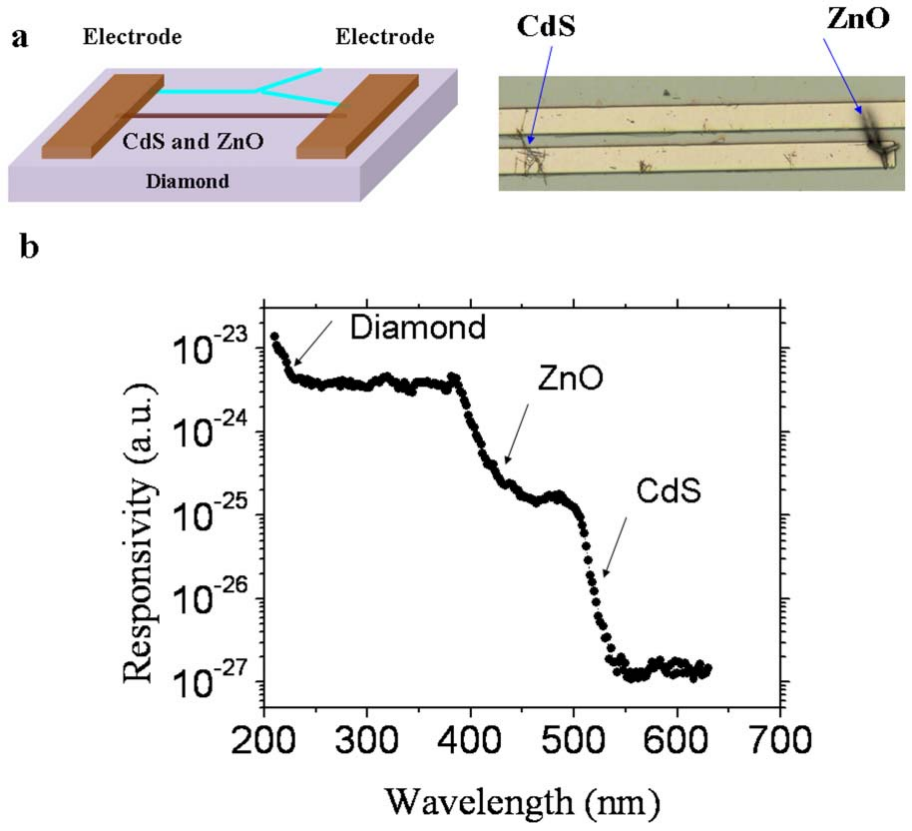
Visible light, UV light, and DUV light three-band photodetector created by integrating ZnO sub-microrods and CdS nanowires on a diamond layer. (a) Schematic showing and optical image the device structure. (b) Spectral response of the two-band photodetectors, demonstrating the three-band response to visible light, UV light, and UV light.

Three-band photodetectors can be developed by integrating two different semiconductor nanostructures on wide-bandgap semiconductors such as diamond or InGaN. CdS (2.5 eV) nanowire and ZnO (3.37 eV) tetrapod-branched nanorod (Supplementary Fig. S4) were integrated on diamond to fabricate three-band photodetectors covering light from the visible region to the UV-A region and to the DUV region, as shown in Fig. 5a. Due to the leakage current from the ZnO-branched sub-microrod, the dark current of the hetero-integrated photodetectors shows an increase to the nanoamper level at a high voltage of 32 V. Due to the high photosensitivity, high photo-to-dark current ratios are achieved upon illumination with visible, UV-A or DUV light. The three-band absorption from the three materials can be distinguished in the photocurrent response spectrum (Fig. 5b), which indicates very good spectral selectivity. As expected, the time response is determined by each semiconductor integrated into the detector (Supplementary Fig. S6, S8, and S10).

## Discussion

The present strategy for multicolor detection has the feature of the simplicity while maintaining the other photoresponse properties. Multicolor photodetection can be simply achieved in a two-terminal device structured like a diode or a metal-semiconductor-metal device. The ultimate performance of the hetero-integrated photodetetor is determined by each semiconductor utilized. In principle, one could achieve the best photoresponse if the best-quality semiconductors are selected for hetero-integration. For example, diamond/Ga_2_O_3_ is the best for solar-blind DUV detector, which possesses the excellent thermal stability under elevated temperatures (Supplementary Fig S.12). The dark current still remained an extremely low level (<pA) for diamond at around 500 K at least. For *β*-Ga_2_O_3_ nanostructures, the photoresponse properties were thermally stable up to 550 K (Fig. S12). The thermal stabily for the InGaN-based photodetector was demonstrated up to 523 K.

The detectivities of multicolor photodetectors can be estimated by taking the real effective absorption area for each material into account. If the shot noise from the dark current is a major contribution, the detectivity can be expressed as^15^

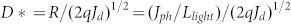
where 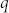 is the absolute value of a single electron's charge, 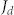 is the dark current, 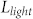 is the intensity of the light, and 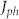 is the photocurrent. For a three-band CdS/ZnO/diamond photodetector, the detectivities are calculated to be 6.34 × 10^14^ Hz^1/2^/W, 5.6 × 10^15^ Hz^1/2^/W, and 3.05 × 10^14^ Hz^1/2^/W under the illumination of 480 nm, 360 nm, and 220 nm light, respectively, at a voltage of 32 V. These detectivities are among the highest for photodetection.

The cut-off bandedge determines the spectral selectivity. Because the photoresponse properties of the hetero-integrated photodetectors using different semiconductor nanostructures depend on each individual material, arbitrary spectral response can be achieved by properly selecting the semiconductors. In principle, no interface states are generated by the integration, the 6S requirements can be readily achieved by controlling each nanostructured semiconductor. Depending on the applications, one can select the proper nanostructures for either full-color detection with wide spectral range or multicolor photodetection with designed spectral selectivity. For example, dual-band detection of DUV and infrared light can be achieved by integrating a narrow bandgap semiconductor (PbS, Si, InGaAs etc nanowires) on diamond or AlGaN substrate. This would show great advantage in improving the signal-to-noise ratio and the warning accuracy in the flame detection such as missile threat. For full-color photodetection, if compositionally tuned nanostructures of In_1-x_Ga_x_N and CdS_x_Se_1-x_ are integrated with a narrow-bandgap semiconductor such as graphene or PbS on a wide bandgap semiconductor like diamond or AlN, BN, the cut-off bandedge problem would be overcome for a wide spectral range.

Table 1 compares the methods for multicolor photodetection. As can be seen, none of the previous strategies could perform photodetection with arbitrary spectral selectivity without degrading other photoresponse properties. For example, the stacking multiple photodiodes could extend the spectral band^2^. However, the sensitivity of the bottom photodiodes will decrease due to the optical absorption by the top photodiodes or the electrodes connected. The nanostructures utilized here have small volumes, which have little influence on the optical absorption of the other semiconductors. Compositionally graded nanowires such as CdS_x_Se_1-x_, In_x_Ga_1-x_N, are promising for multicolor photodetectors^18^,^19^. However, one material system can be used only for a specific spectral range. For example, the CdS_x_Se_1-x_ nanowires devices were only for visible light detection^19^. Actually, the reported CdS_x_Se_1-x_ photodetector was basically an individual device for a certain optical band. While In_x_Ga_1-x_N is potentially for multicolor photodetector from IR to UV, the practical devices based on In_x_Ga_1-x_N for IR is a challenge up to now. This is because that the reported InN exhibits metallic conductivity in actual case^34^. In addition, DUV detection can not be achieved by using In_x_Ga_1-x_N limited by the bandgap of GaN. Therefore, it is still impossible to use only one material system with graded composition for multicolor photodetection covering arbitrary spectral band. The organic semiconductor can be applied for full-color photodetection with wide spectral range from 300–1450 nm^15^. However, in some cases like flame detection, photoresponse to visible light is a serious noise, when spectral selectivity on DUV and IR bands is required. Moreover, organic materials are notoriously unstable, especially under extreme conditions such as high temperatures and high incident light power. Quantum structures such as stacked Al_x_Ga1-_x_N hetero-junction are always applied for dual-band photodetection^35^. However, complex fabrication process is in demand for obtaining the sharp interface. Although coupling the semiconductor with plasmonic nanostructures provides an alternative strategy to extend the spectral range in the visible range^11^, the accurate control is quite challenge, especially for much wider spectral band.

We mention that for the response speed in Table 1, persistent photoconductivity^36^ rather than “fast” or “ slow” is utilized. The response speed of a photodetector strongly depends on the light intensity since there are more or less defects in a semiconductor. Under strong light illumination, band (valence) to band (conduction) recombination would determine the ultimate response time. In such a case, the response speed would be very fast. For example, our diamond photodetector under strong excimer 193 nm light illumination could follow well the pulse width of the laser with a time of 10 ns^37^. Under weak DUV light illumination from a Xe light source, defects would affect the response speed obviously. The decay time would be much longer. Therefore, persistent photoconductivity seems more accurate to describe the response speed of a photodetector.

The present strategy for multicolor photodetection provides significant freedom for arbitrary spectral band with high photoresponse performance. Notable progress in the preparation of various semiconductor nanostructures allows for fabrication of photodetectors sensitive to any optical spectral band. The integration of different semiconductor nanostructures on the same wide-bandgap semiconductor is also a universal strategy for the development of other energy-conversion optoelectronic devices, such as full-spectrum solar cells, light-emitting diodes, and photochemical catalysts.

## Methods

The materials preparation and characterization are detailed in Supporting Information I. Single-crystal diamond layer and InGaN layers were grown by chemical vapor depositions. Semiconductor nanomaterials were prepared by means of high-temperature reaction or hydrothermal reaction, and then were characterized using an X-ray powder diffractometer (Rigaku Co., Japan), a scanning electron microscopy (S-4800), and a transmission electron microscopy (JEM-2100F, equipped with an energy-dispersive X-ray spectrometer).

The interdigitated metal-semiconductor-metal (MSM) configuration was utilized for the photodetector device fabrication by using a standard photolithography process. The semiconductor nanostructures were placed on the surface of the diamond epilayer or the InGaN thin film. The finger width and spacing are both 10 μm. A thin Ti layer with a thickness of 40 nm followed by a tungsten carbide (WC) cap layer with a thickness of 30 nm was subsequently deposited on the patterned samples as the metal contacts.

The current-voltage (I–V) characteristics were measured using a two-point probe method with an Advantest picoammeter (R8340A) and a dc voltage source (R6144). Either dc current mode or lock-in amplifier technique was employed to record the spectral response, in which a 500-W Ushio xenon lamp and an Acton Research monochromator with order sorting filters were used. The light intensity was modulated through an aperture and calibrated by using a UV-enhanced Si photodiode. The spectral response was measured by a standard lock-in amplifier technique with a chopped frequency of 100 Hz. The lock-in measurement ensures here that the shape of the spectral response does not depend on the light intensity. The ultimate photoresposne spectra were normalized by using the light flux of the Xeon lamp illuminated on the samples by each wavelength.

## Author Contributions

M.Y.L., L.W.S. and J.Q.H. conceived the idea. L.W.S. and M.Y.L. did the device fabrication and photocurrent measurements. R.J.Z. and J.Q.H. grew all the nanomaterials. L.W.S., M.Y.L. and J.Q.H. analyzed the data. L.W.S., M.Y.L. and J.Q.H. wrote the main manuscript text. Y.K. discussed the content. All authors reviewed the manuscript.

## Supplementary Material

Supplementary Information Arbitrary Multicolor Photodetection by Hetero-integrated Semiconductor Nanostructures

## Figures and Tables

**Table 1:** Comparison among the methods for multicolor photodetection

	Methods					
6S parameter	Hetero-integration	Multiple photodiode	Alloys	Organic Semiconductor	Graphene + plasmon	Hetero-junction
Quantum efficiency (%)*1	>100	NA	NA	<100	<100	<100
**Spectral selectivity**	Arbitrary	Three band	One band, adjustable	Multicolor (300–1450 nm)	Multicolor (450–600 nm)	Dual band (DUV, UV)
Persistent photoconductivity*2	Little	NA	Little	NA	NA	NA
Dark current/density*3	<10^−12^A	10^−3^A	10^−9^A	10^−5^A/cm^2^	10^−9^A	10^−12^A
**Stability**	Good	Good	NA	No	NA	NA
**Simplicity**	Yes	No	NA	Yes	NA	No
**References**	This work	2	19	15	16	35

*^1^Quantum is used to evaluate the sensitivity.

*^2^Persistent photoconductivity is to evaluate the response speed.

*^3^Dark current/density is assumed to represent the signal-to-noise ratio.
