# Supplementary material for: Arbitrary Multicolor Photodetection by Hetero-integrated Semiconductor Nanostructures
Source: Sci Rep. 2013 Aug 6;3:2368. doi: 10.1038/srep02368 (PMC3734442; doi:10.1038/srep02368)
Supplement: Supplementary Information — Arbitrary Multicolor Photodetection by Hetero-integrated Semiconductor Nanostructures [file srep02368-s1.pdf]

Supplementary Information

Arbitrary Multicolor Photodetection by Hetero-integrated Semiconductor Nanostructures

Liwen Sang[1,2], Junqing Hu[2*], Rujia Zou[2], Yasuo Koide, [1] and Meiyong Liao[1]*

[1] National Institute for Material Science (NIMS), 1-1 Namiki, Tsukuba, Ibaraki, 305-0044, Japan. [2]State Key Laboratory for Modification of Chemical Fibers and Polymer Materials, College of Materials Science and Engineering, Donghua University, Shanghai 201620, China, *e-mail: hu.junqing@dhu.edu.cn; meiyong.liao@nims.go.jp

I. Materials preparation and characterization

All of the chemicals used for the material preparation are commercially available and were used as received without further purification. Sn powders, Zn plate, GaN powders, $Fe(NO_3)_3$, $Cd(CH_3COO)_2$•$2H_2O$, ethylenediamine, ethanol, acetone, methane, trimethylgallium, trimethylindium, and ammonia were purchased from Sinopharm Chemical Reagent Co.

1. Intrinsic single-crystal diamond layer

The single-crystal diamond layer was homoepitaxially grown on a high-pressure, high-temperature type-Ib (100) single-crystal diamond substrate by using a microwave plasma-enhanced chemical vapor deposition reactor. The diamond substrate was treated by boiling a solution of H_2SO_4/HNO_3 for 3 hours. The fed gases for the growth of diamond were H_2 and CH_4. The CH_4/H_2 flow ratio during the growth was 0.08-2%. The substrate was heated to 900-1000 ^oC by the plasma during the growth. The total pressure of the chamber during the growth was maintained at 80

Torr. The crystal quality was confirmed by various techniques such as Raman, catholuminescence[1,2].

2. InGaN layer

The InGaN thin film was epitaxially grown on *c*-plane sapphire substrate by metalorganic chemical vapor deposition. Trimethylgallium ($(CH_3)_3Ga$), trimethylindium ($(CH_3)_3In$) and ammonia (NH_3) were used as the precursors. Initially, the sapphire substrate was heated up to 1050 ^oC for 20 min in hydrogen ambient, followed by the deposition of 25-nm-thick GaN buffer layer at 500 ^oC. Then the temperature was raised to 1000 ^oC to grow 1.5-μm-thick GaN epilayer. Finally, the substrate temperature was reduced to 800 ^oC and InGaN film with the thickness of 300 nm was deposited. The high quality of the InGaN thin film was confirmed by high-resolution X-ray diffraction (HRXRD) measurement using a Panalytical Xpert PRO XRD system[3].

3. Ga_2O_3 nanobelts

β-Ga_2O_3 nanobelts were grown in horizontal high-temperature resistance alumina tube furnace. GaN powders (0.2 g) were put into an alumina boat, which was placed at the central region of an alumina tube. A long alumina plate, ultrasonically cleaned in acetone, was used as substrate, which was inserted downstream into the tube. The alumina tube was then sealed and a constant gas flow of Ar mixed with 5% H_2 was introduced through the tube at a flow rate of 150 sccm and under ambient pressure. The furnace was heated at an elevating temperature speed of 10 ^oC/min to 900 ^oC and was kept at this temperature for 1.0 h, and then increased to 1200 ^oC at the same speed and stably maintained at this temperature for 3.0 h. After the furnace was cooled to room temperature naturally, the products were collected from the alumina substrate for following experimental use.

Fig. S1a shows comparative XRD patterns of as-synthesized β-Ga_2O_3 nanobelts (top) and the standard β-Ga_2O_3 powders (bottom) from the JCPDS file (card no.: 43-1012), respectively. All reflection peaks in the XRD pattern of the products can be readily

indexed with the sequence of reflection of the monoclinic β-Ga_2O_3 phase with lattice parameters a = 1.223 nm, b = 0.304 nm, and c = 0.580 nm, and β = 103.7 ° without any characteristic peaks from other crystalline forms. A typical SEM image, Fig. S1b, reveals that the products consist of a large quantity of belt-like or sheet shaped morphologies. Among these products, the length is up to tens of micrometers, and the width is in the range of several hundreds of nanometers to tens of micrometers and the thickness is tens of nanometers. TEM image, Fig. S1c, shows that the nanobelt has a uniform width along its length and displays the smooth surface. A high resolution TEM (HRTEM) image of a β-Ga_2O_3 belt in Fig. S1d reveals lattice fringes with a d-spacing of 0.21 nm and 0.20 nm, which match that of the (-112) and (600) planes of the β-Ga_2O_3 crystal, respectively. The belt is a structurally-uniform single crystal, and no dislocations or other planar defects are observed within it; also, the edge of the belt is clean and abrupt on an atomic scale, and there are no amorphous layers covering the surface. An upper-right inset in Fig. S1d shows the corresponding fast Fourier transformation (FFT) pattern of the image, which can be indexed to the [0-21] zone axis of the β-Ga_2O_3 crystal.

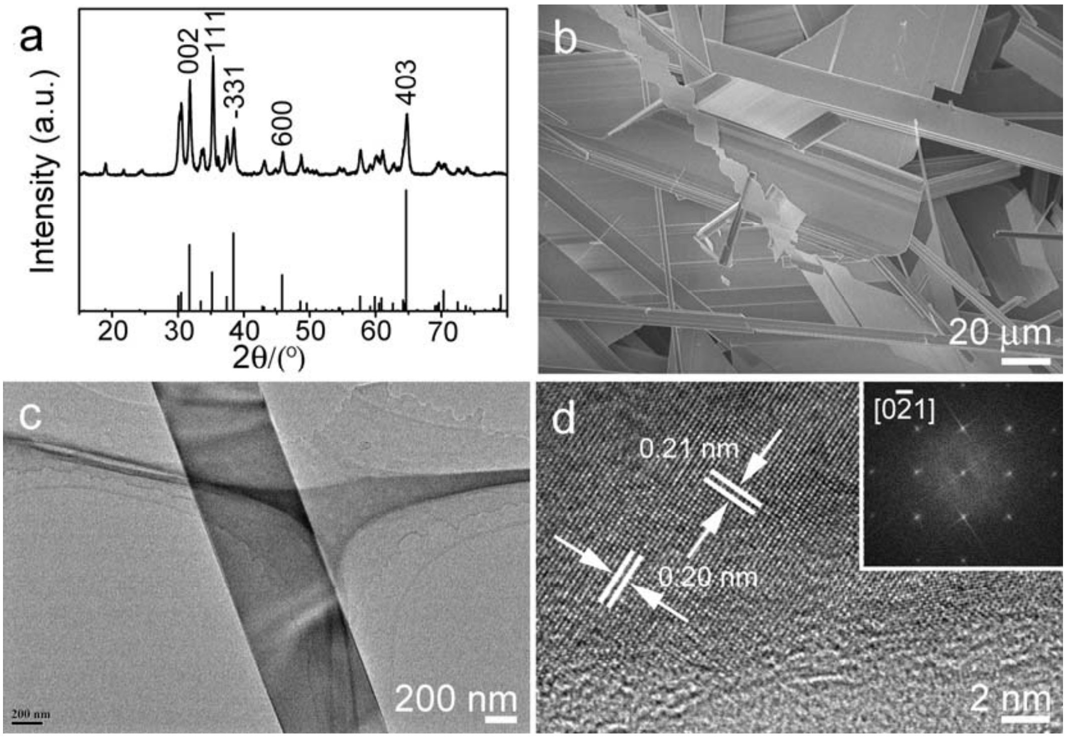

Fig. S1. The synthesis and characterization of *β*-Ga_2O_3 nanobelts. a, XRD patterns of as-synthesized *β*-Ga_2O_3 nanobelts (top), by referring to the standard *β*-Ga_2O_3 powders from the JCPDS files (card no.: 43-1012) (bottom). **b, c,** and **d,** SEM, TEM and HRTEM images of the synthesized *β*-Ga_2O_3 nanobelts, respectively, an upper-right inset in (d) showing the FFT pattern along the [0-21] zone axis of the *β*-Ga_2O_3 crystal.

4. SnO_2 nanobelts

The SnO_2 nanobelts were synthesized through thermal oxidation reactions in a horizontal high-temperature resistance alumina tube furnace[4]. A mixture of Sn (3 g) and $Fe(NO_3)_3$ (5 g) powders were placed on an alumina wafer, which was placed at the center of the alumina tube. The tube was sealed and then evacuated by a mechanical rotary pump to a pressure of 6×10^{-2} Torr. During the experiment, a constant flow of Ar mixed with 5% H_2 was maintained at a flow rate of 50 sccm, and the pump continually evacuated the system so that the pressure inside the tube was kept at 350 Torr. The temperature of the furnace was increased to 800 °C from room temperature at an elevating temperature speed of 30 ^{o}C/min and was kept at 800 °C for 30 min and then further increased to 1080 °C at the same speed and maintained at this temperature for 30 min. After the furnace was cooled to room temperature, white wool-like products were formed on the inner wall of the tube near the cooling finger.
Fig. S2a shows XRD pattern of as-synthesized SnO_2 nanobelts (top) as referenced by standard SnO_2 phase from JCPDS files (card no.: 41-1445) (bottom). All diffraction peaks of as-prepared products can be indexed to a tetragonal rutile structure of SnO_2 material with lattice constants of a = 4.75 and c = 0.320 nm, in agreement with the reported values (a = 0.4738 and c = 0.3187 nm) from the JCPDS files. No other peaks associated with other impure crystalline forms were detected in the pattern. SEM image, Fig. S2b, shows that as-synthesized products consist of a large quantity of straight nanobelts; the lengths and thicknesses of the belts have a size distribution of several hundreds of nanometers to tens of micrometers and 10-30 nm, respectively. TEM image, Fig. S2c, reveals that each belt has a uniform width along its entire

length, and the typical width is in the range of 100-300 nm. As seen from a HRTEM image, Fig. S2d, the resolved lattice fringes with a d-spacing of 0.24 nm and 0.24 nm matches that of the (200) and (020) lattice planes of the SnO_2 crystal, respectively. Further analysis suggests that this belt is a single-crystal in nature and grew along the [100] direction, and the belt edge is clean and abrupt on an atomic scale and there are no amorphous layers covering the surface. An upper-right inset shows the corresponding FFT pattern of image, which can be indexed as the [001] zone axis of the SnO_2 crystal.

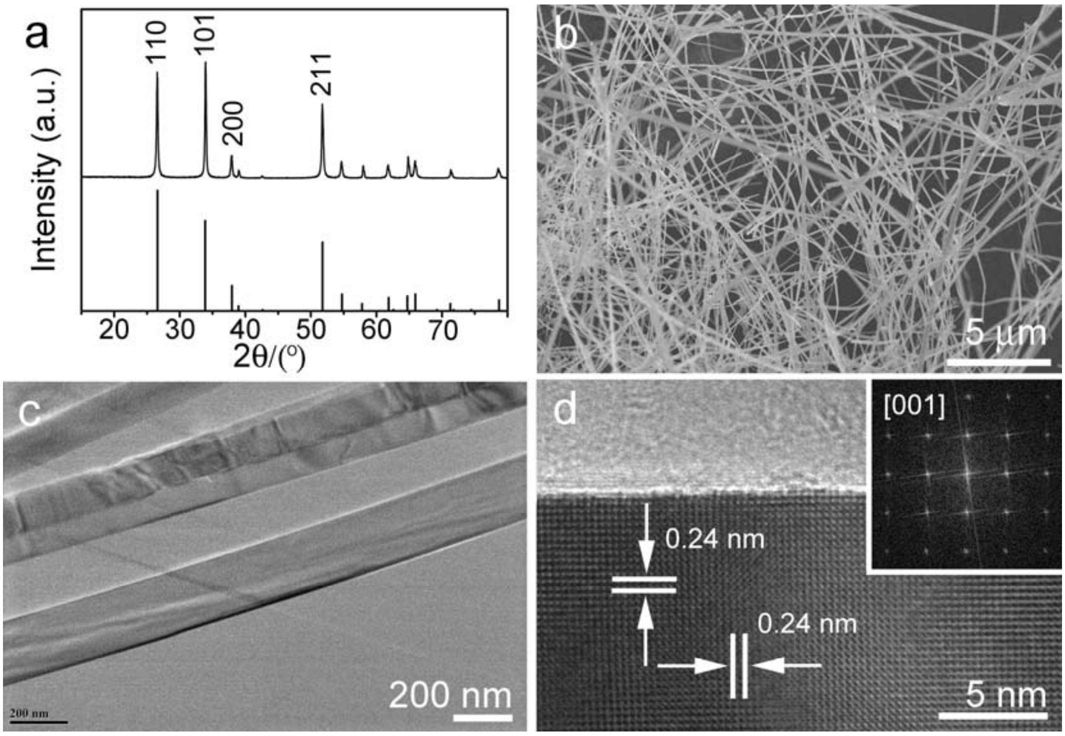

Fig. S2. The synthesis and characterization of SnO_2 nanobelts. a, XRD pattern of as-synthesized SnO_2 nanobelts (top), as referenced by standard SnO_2 powders from the JCPDS files (card no.: 41-1445) (bottom). **b, c** and **d,** SEM, TEM and HRTEM images of the SnO_2 nanobelts, respectively, an upper-right inset in (d) showing the FFT pattern along the [001] zone axis of the tetragonal SnO_2 crystal.

5. CdS nanowires

The CdS nanowires were synthesized through a simple hydrothermal reaction. In a

typical synthesis, S powders (0.064 g) and $Cd(CH_3COO)_2$•$2H_2O$ (0.267 g) were dissolved in 50 mL of ethylenediamine under vigorous stirring and then transferred into a 60 mL Teflon-lined autoclave with a stainless-steel shell. The autoclave was heated to 220 °C for 48 h and then cooled to room temperature naturally. As-obtained products were washed successively with deionized water and ethanol to remove any residual ionic species. After being dried in a vacuum at 60 °C for 12 h, the final products were characterized for following experimental use.

Fig. S3a displays comparative XRD patterns of as-grown CdS nanowires (top) and the standard CdS powders from the JCPDS files (card no.: 41-1049) (bottom), respectively. All of the peaks of the synthesized products can be readily indexed according to the hexagonal (wurtzite) phase of CdS with lattice constants of a = 0.415 and c = 0.670 nm, in agreement with the reported values (a = 0.4141 and c = 0.672 nm) from the JCPDS card. A low-magnification SEM image, Fig. S3b, shows that the products consist of a large quantity of straight nanowires with a length up to tens of micrometers. A TEM image, Fig. S3c, reveals that each CdS nanowire has a uniform diameter along the length, and most of them have a diameter of ~ 80-100 nm. A high resolution TEM image, Fig. S3d, confirmed the structural uniformity of this single-crystal CdS nanowire; the lattice fringes of the (100) and (001) planes with a *d*-spacing of 0.36 and 0.67 nm, respectively, can be clearly seen, and the wire's axis direction, i.e., the growth direction, was parallel to the [001] crystallographic orientation of a wurtzite CdS crystal. An upper-right inset shows the corresponding FFT pattern of image, which can be indexed as the [100] zone axis of the hexagonal CdS crystal.

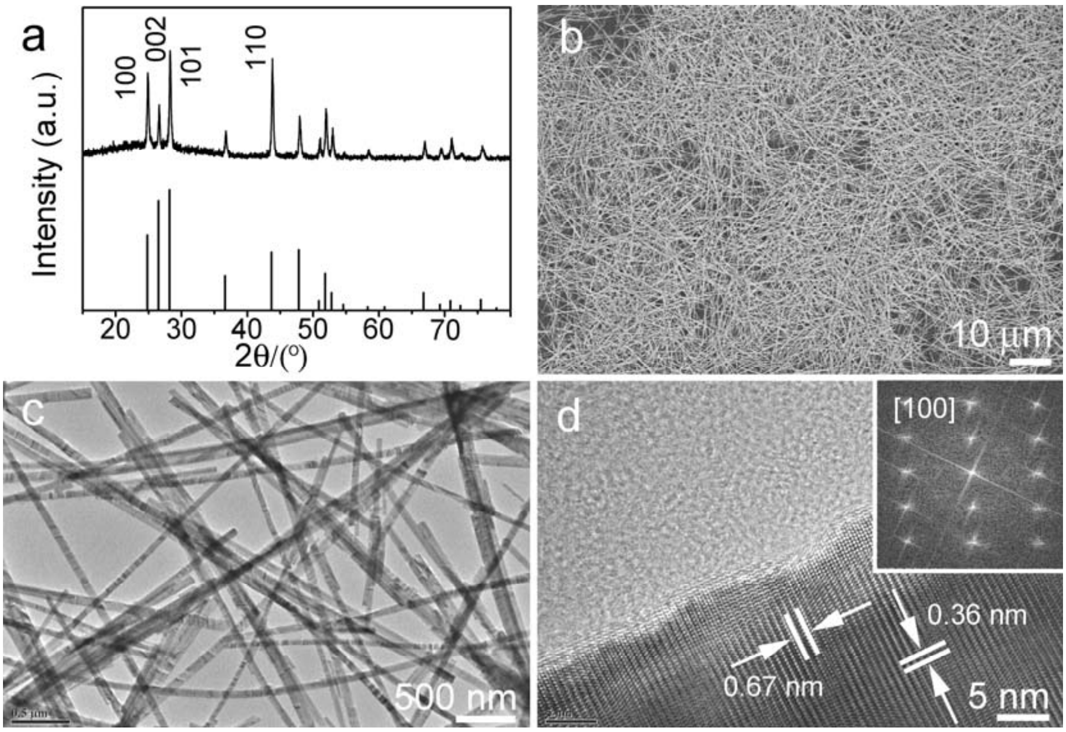

Fig. S3. The synthesis and characterization of CdS nanowires. a, XRD patterns of as-synthesized CdS nanowires (top) and the standard CdS powders from the JCPDS files (card no.: 41-1049) (bottom). **b, c,** and **d,** SEM, TEM and HRTEM images of the CdS nanowires, respectively, an upper-right inset showing the FFT pattern along the [100] zone axis of the CdS crystal.

6. Tetrapod branched ZnO sub-microrods

The tetrapod branched ZnO sub-microrods were synthesized through a simple evaporation and oxidation method in air atmosphere. A Zn plate (99%), which was used as a source material, was ultrasonically cleaned in acetone, and then was placed inside an alumina crucible. The alumina crucible containing this Zn plate without lid was inserted into the center of the furnace. The furnace was heated to 1200 °C at an elevating temperature speed of 10 °C/min and was maintained at this temperature for 2.0 h during the oxidation, and then cooled down to room temperature, naturally. The white products were collected from the crucibles for following experimental use.

Fig. S4a shows the XRD pattern of as-synthesized ZnO products (top), which was referred to the XRD pattern of the standard ZnO powder of the JCPDS files (card no.:

36-1451) (bottom). Clearly, the XRD pattern of the products is in accordance with those of the wurtzite structure of the ZnO crystalline powders and no any peaks from impurities such as Zn are found. A low-magnification SEM image, Fig. S4b, displays that the synthesized products are entirely composed of tetrapod-shaped ZnO crystals with four branches extending from a common center. Each branch within an architecture appears like a needle-shape with the diameter gradually decreasing from the root (or common center) along the length (~ 6-10 μm) forming a rather sharp tip. Fig. S4c is a TEM image of such a tetrapod-shaped ZnO crystal, which demonstrates the same four-branched morphological characteristic as the observation from the SEM imaging. Fig. S4d is a HRTEM image taken from the tipped part of a branch within the tetrapod-shaped ZnO crystal; it shows clearly hexagonal lattice characteristic of the ZnO crystal; the lattice spacing of 0.52 nm between adjacent lattice planes corresponds to the distance between two (001) crystal planes, indicating the preferential growth of the branch along the [001] direction.

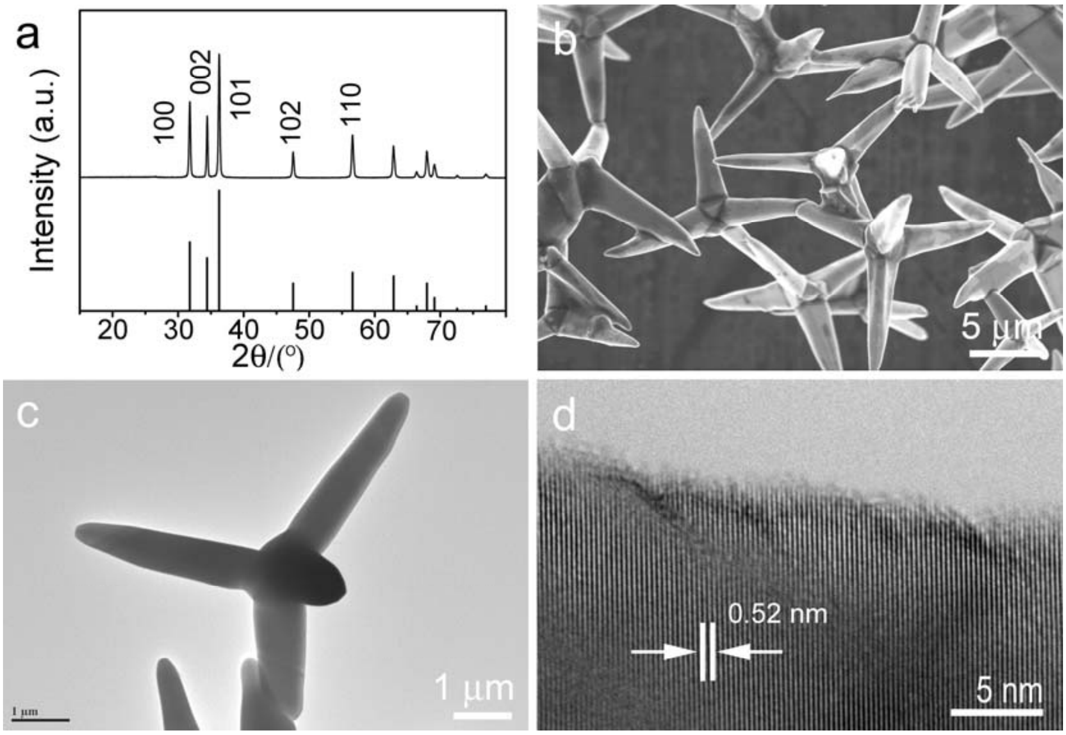

Fig. S4. The synthesis and characterization of tetrapod-branched ZnO sub-microrods. a, XRD patterns of as-synthesized tetrapod-branched ZnO products (top) and the standard ZnO powders from the JCPDS files (card no.: 36-1451)

(bottom). **b,** SEM image of the synthesized tetrapod-branched ZnO crystals. **c** and **d,** TEM and HRTEM images of a tetrapod-branched ZnO crystal, respectively.

II. Devices fabrication and photoresponse properties measurements

1. Single-crystal diamond layer

Diamond has been demonstrated to be a promising material for solar-blind photodetectors[5-8]. The dark current of the intrinsic was extremely low (< 10^{-13} A) at the applied voltage up to 32 V, indicative of the high resistivity of the diamond epilayer. The photoresonse properties of the diamond layer grown on the type Ib (100) diamond substrate are illustrated in Fig. S5. A significant photocurrent was observed when the diamond was exposed to the DUV 220 nm light (Fig. S5a). The quantum efficiency of the diamond photodetector upon 220 nm light illumination depends on the applied voltage[8]. Little response was observed in the visible light region (Fig. S5b), revealing a high DUV/visible light rejection ratio. The cut-off wavelength is located at 225 nm, corresponding to diamond band edge. The intrinsic diamond layer also displays a fast response (Fig. S5c).

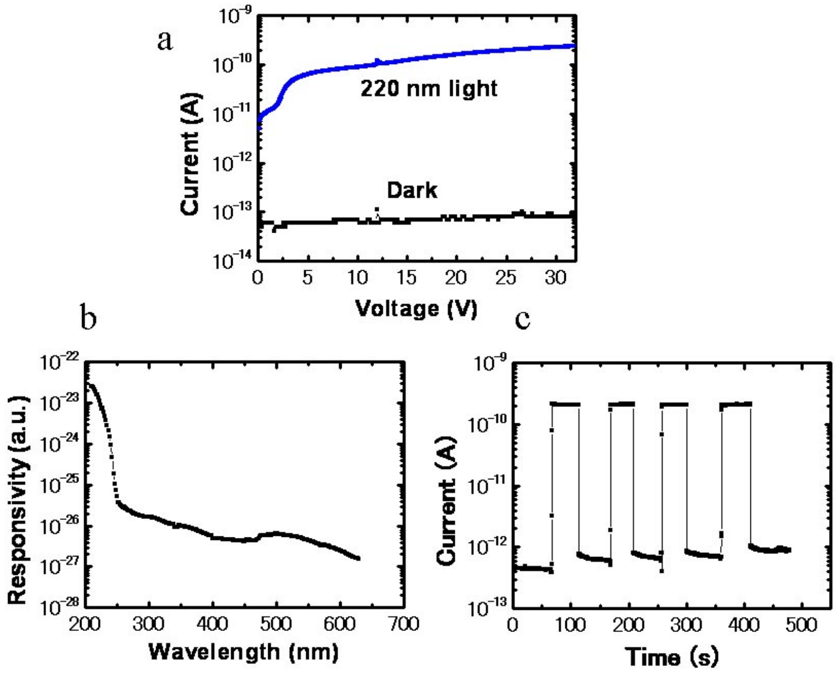

Fig. S5. Typical photoresponse properties from the intrinsic diamond layer. a, Current-voltage characteristic in dark and upon the 220 nm light illumination with a

density of 20 μW/cm^2. **b,** Spectral response of the intrinsic diamond layer at a bias of 5 V. **C,** Time response disclosing a fast response upon the 220 nm light ON/OFF switching.

2. InGaN layer

III-Nitride semiconductor InGaN, with the tunable direct bandgap energy, high theoretical responsivity, high breakdown voltage, and sharp cutoff wavelength, offers an alternative and potentially better approach for detecting ultraviolet-A (UV-A) light[9]. By using a superwide bandgap insulator of calcium fluoride (CaF_2) as metal-insulator-semiconductor structure, a very low dark current of 1.47×10^{-10} A was obtained, which makes it suitable for the hetero-integration with semiconductor nanostructures for multi-band photodetectors (Fig. S6). The photocurrent-to-dark current ratio of the InGaN photodetector is more than 10^5 upon 338 nm illumination at a bias voltage of 2 V, as shown in Fig. S6a. Due to the great decrease of the leakage current, the InGaN photodiodes show a high discrimination ratio more than six orders of magnitude between UV and visible light, which is the largest value reported for the GaN-based photodiodes up to now (Fig. S6b). The device also displays a fast response speed, with little persistent photoconductivity (Fig. S6c), and a response time of 747 μs under the bias voltage of 0.1 V.

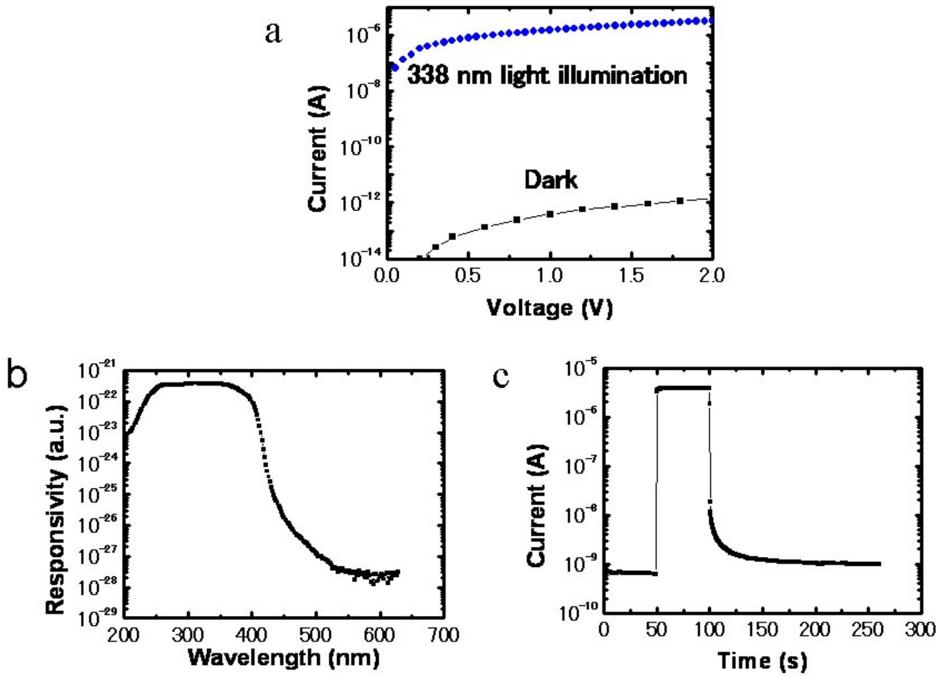

Fig. S6. Typical photoresponse properties from the InGaN thin film. a, *I-V* characteristics and responsivity of InGaN photodector in the dark and upon 338 nm light illumination. **b,** Photocurrent spectra measured under the illumination of xenon lamp at the applied voltage of 1 V. **c,** Time response upon 350 nm illumination measured by a mechanical chopping method.

3. Ga_2O_3 nanobelts

With a bandgap of ~ 4.9 eV, β-Ga_2O_3 nanobelts are an ideal candidate for solar-blind DUV-light sensors and optoelectronic circuits[10,11]. Fig. S7a shows the *I-V* curves of the β-Ga_2O_3 nanobelt photodetector exposed to 250 nm light and under dark condition. A high photoexcited current larger than 50 nA was recorded at a bias of 32 V. The corresponding logarithmic plot clearly shows that the photo-excited current significantly increases by six orders of magnitude larger than the dark current ($<10^{-13}$ A) at the same conditions, indicating a high signal-to-noise ratio. The photoresponsivity at 250 nm is almost 6 orders of magnitude larger than that in the visible light region (Fig. S7b). The cut-off wavelength is located at 250 nm. The Ga_2O_3 nanobelts also display a fast response, as shown in Fig. S7c. The ON and OFF

state currents for each cycle shown in the figure remain the same level, indicating the high reversibility and high stability.

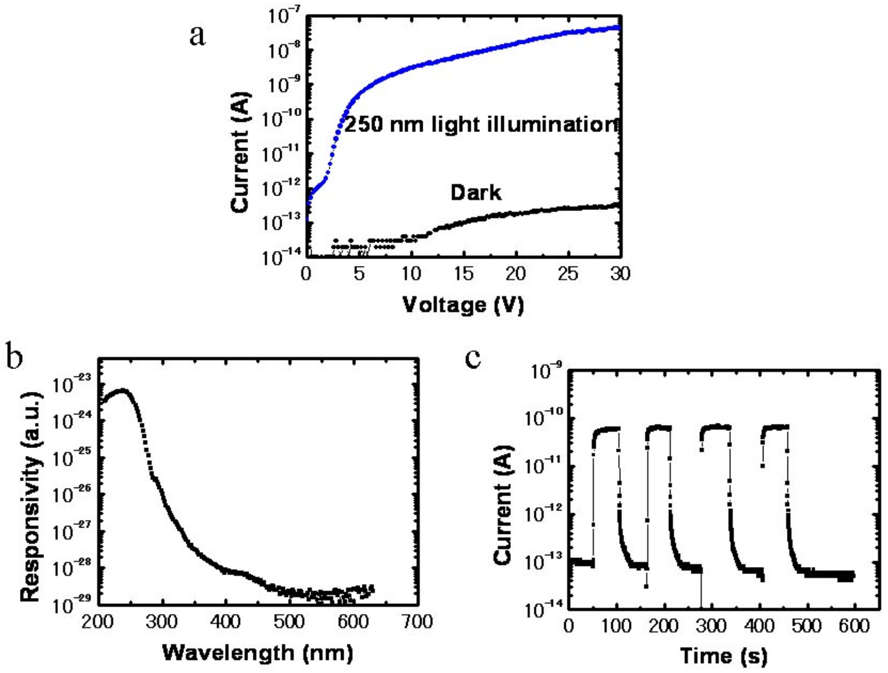

Fig. S7. Photoresponse properties from the β**-**Ga_2O_3 **nanobelt. a,** *I-V* curves of the β-Ga_2O_3 nanobelt photodetector in dark (black curve) and under 250 nm light (blue curve) illumination. **b,** A photoresponse of as-fabricated β-Ga_2O_3 nanobelt photodetector measured at a bias of 5 V under various wavelengths from 210 nm to 630 nm. **C,** Time response of the β-Ga_2O_3 nanobelt photodetector illuminated by 250 nm light at a bias of 5 V.

4. CdS nanowires

As an important II-VI group semiconductor, CdS nanowires deserve particular attention owing to their wide optoelectronic applications for visible-light photodetecting in the green light region (E_g=2.42 eV)[12,13]. The photoresponse properties of the CdS nanowires are illustrated in Fig. S8. The dark current of the CdS photodiodes is as low as 8.5 pA at the applied voltage of 32 V, thus a significant increase of the photocurrent of more than 10^3 was observed when the CdS nanowires were exposed to the 480 nm visible light illumination (Fig. S8a). The low dark current also brings forward a high discrimination ratio of more than 100 between green light

and red light (Fig. S8b). The CdS nanowires also display a fast response speed, as shown in Fig. S8c, in which little persistent photoconductivity was observed.

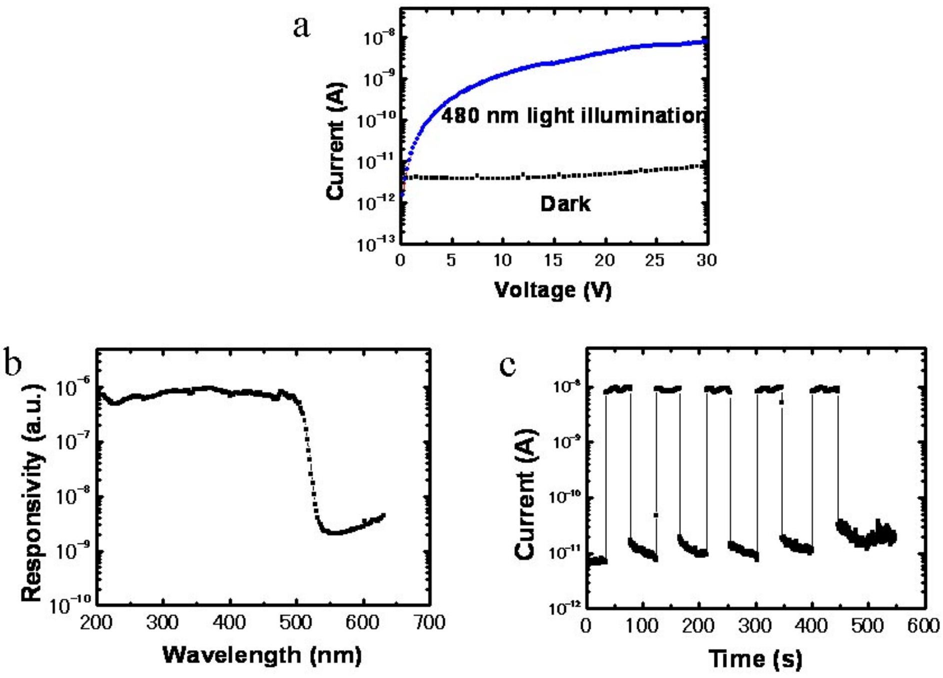

Fig. S8. Photoresponse properties from the CdS nanowire. a, *I-V* characteristics of CdS nanowire photodetector in the dark (black curve) and upon 480 nm (blue curve) light illumination. **b,** Photocurrent spectra measured under the illumination of xenon lamp at the applied voltage of 16 V. **c,** Time response upon 480 nm illumination measured by a mechanical chopping method at the applied voltage of 32 V.

5. SnO_2 nanowires

Tin dioxide (SnO_2) with a wide direct bandgap in the ultraviolet region and high quantum efficiency in the ultraviolet region, has attracted a great deal of attention as a good material for visible-blind photodetection[14,15]. Due to the intrinsic *n*-type conduction, the dark current of the SnO_2 nanowires is relatively high. Fig. S9a indicated the dark current and photocurrent under 280 nm light illumination of the fabricated SnO_2 nanowire photodetectors with the applied voltage. As can be seen, even if the SnO_2 nanowires illustrated a high dark current up to the order of 10^{-7} A, the photocurrent was still ten times higher. Compared with the reported SnO_2 nanowire photodetectors, our developed device showed a better spectrum selectivity[16],

as shown in Fig. S9b. The photodetector displayed a cutoff from 300 nm and the UV/visible discrimination ratio between 280 nm and 600 nm is more than 10^3.

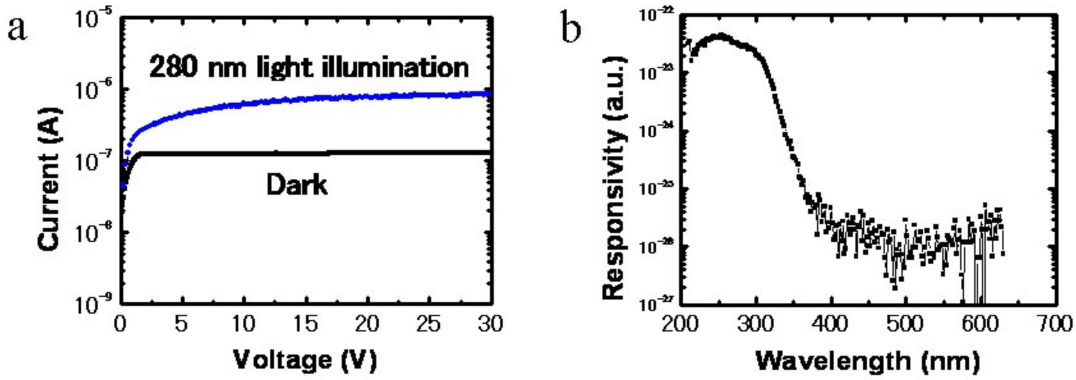

Fig. S9. Photoresponse from SnO_2 nanowire. a, *I-V* characteristics of SnO_2 nanowire photodetector in the dark (black curve) and upon 480 nm light (blue curve) illumination. **b,** Photocurrent spectra measured under the illumination of xenon lamp at the applied voltage of 2 V.

6. Tetrapod-like ZnO sub-microrods

Because of its wide band gap (E_g=3.4 eV), low cost, and easily manufacturing, ZnO has been emerging as a potential candidate in optoelectronic applications, including light-emitting diodes, laser diodes, and photodetectors for the UV spectral range[17]. In the past decade, ZnO nanowire devices have demonstrated good performance for the UV photodetectors[18]. However, due to the large surface-to-volume ratio and the presence of deep level surface trap states, the ZnO nanowire devices always suffer from a big leakage current. In this work, the tetrapod-branched ZnO sub-microrods were synthesized through a simple evaporation and oxidation method in air atmosphere. With this kind of shape, the ZnO photodetectors displayed a low dark current of ~10^{-13} A at an applied voltage as high as 32 V. The photocurrent under 360 nm illumination was 6 orders of magnitude higher than the dark current (Fig. S10a). The UV/visible discrimination ratio was more than 10^4 (Fig. S10b). The branched ZnO sub-microrod devices also showed a fast response speed, and just a little persistent photoconductivity was observed at the

applied voltage of 5 V (Fig. S10c). These results indicated the tetrapodal ZnO sub-microrods could provide a best solution for the high-performance UV photodetection which can satisfy the *5S* requirement.

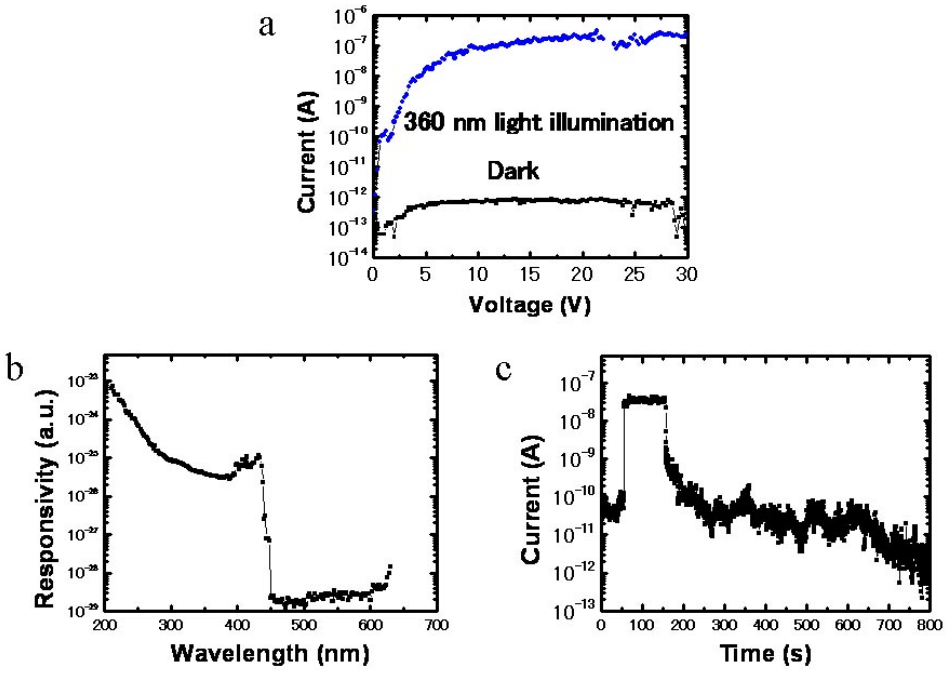

Fig. S10. Photoresponse properties from Tetrapod-like ZnO sub-microrods. a, *I-V* characteristics of the branched ZnO sub-microrod photodetector in the dark (black curve) and upon 360 nm light (blue curve) illumination. **b,** Photocurrent spectra measured under the illumination of xenon lamp at the applied voltage of 16 V. **c,** Time response upon 360 nm illumination measured by a mechanical chopping method at the applied voltage of 5 V.

7. SnO_2/diamond dual-band photodetectors

By using this novel hetero-integration method, any multi-band photodetectors with any expected wavelengths can be realized in principle. Here, SnO_2/diamond two-band photodetectors were fabricated. The spectral response of the developed device at a bias of 32 V was displayed in Fig. S11. The two-band responses located at 220 nm (diamond) and 330 nm (SnO_2 nanowire) were clearly distinguished. At 32 V, the spectral discrimination ratio between DUV (210 nm) and visible light (630 nm)

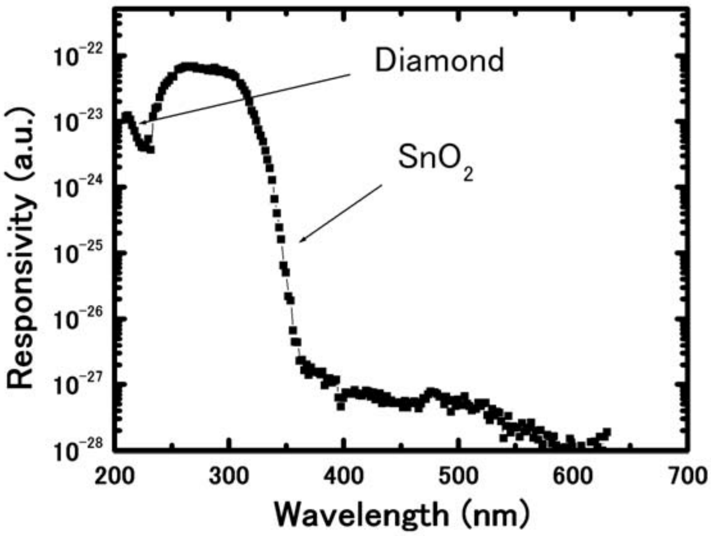

Fig. S11. Photocurrent spectrum of as-fabricated SnO_2/diamond dual-band photodetector measured under the illumination of xenon lamp at the applied voltage of 32 V.

8. Thermally stability of the hetero-integrated photodetectors

As examples to show the stability of the hetero-integrated photodetectors, the dark currents of diamond, InGaN, and the β-Ga_2O_3 detector were investigated up to 550 K. As shown in Fig S 12, the dark currents increase little, which basically does not degrade the overall photoresponse properties of the hetero-integrated photodetectors.

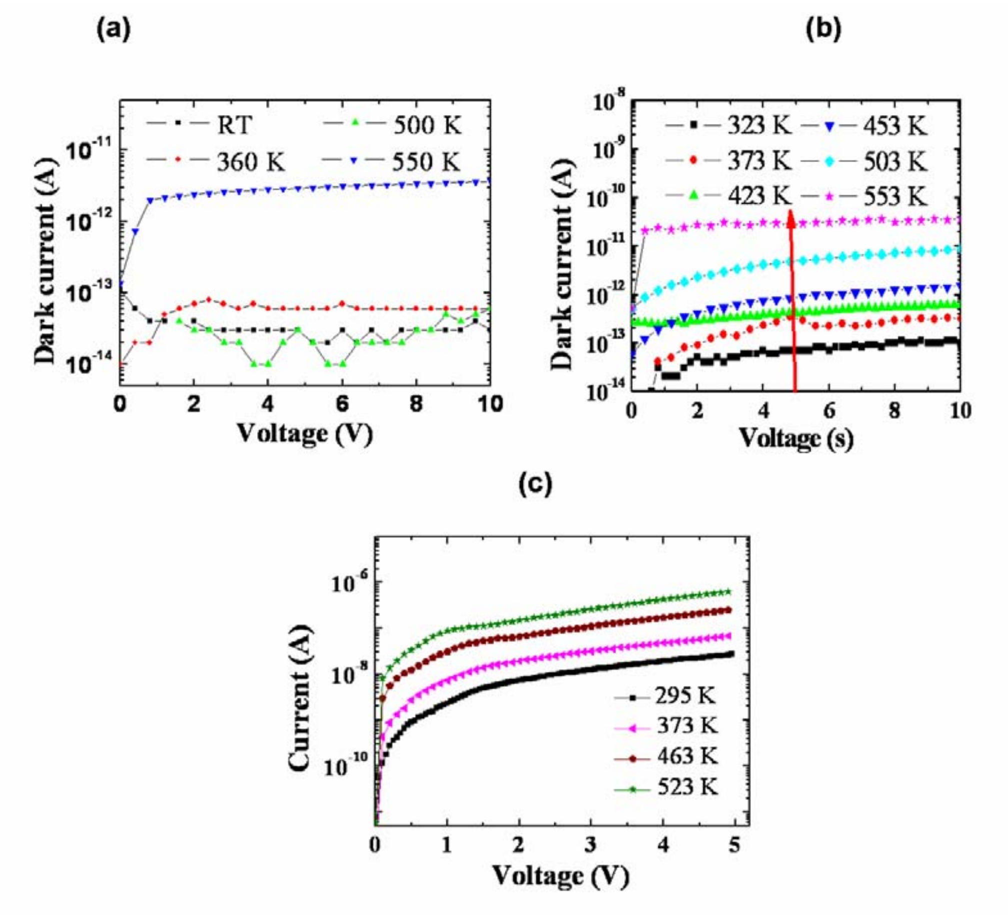

Fig. S12. Dark currents at elevated temperatures for (a) diamond, (b) *β*-Ga_2O_3, and (c) InGaN.

References

1. Aharonovich, I. *et al.* Homoepitaxial growth of single crystal diamond membranes for quantum information processing. *Adv. Mater.* **24**, 54-59 (2012).

2. Okushi, H. High quality homoepitaxial CVD diamond for electronic devices. *Diamond Relat. Mater.* **10**, 281-288 (2001).

3. Sang, L. W., Liao, M. Y., Koide, Y. & Sumiya, M. High-performance metal-semiconductor-metal InGaN photodetectors using CaF_2 as the insulator. *Appl. Phys. Lett.* **98**, 103502 (2011).

4. Hu, J. Q. *et al.* Large-scale rapid oxidation synthesis of SnO_2 nanoribbons. *J. Phys. Chem. B* **106**, 3823-3826 (2002).

5. Liao, M. Y., Alvarez, J. & Koide, Y. Submicron MSM photodiodes toward improving the responsivity. *Appl. Phys. Lett.* **91**, 163510 (2007).

6. Liao, M. Y., Alvarez J. & Koide, Y. Single schottky-barrier photodiode with

interdigitated finger geometry: Application to diamond. *Appl. Phys. Lett.* **90**, 123507 (2007).

7. Liao, M. Y. & Koide, Y. High-performance metal-semiconductor-metal deep-ultraviolet photodetectors based on homoepitaxial diamond thin film. *Appl. Phys. Lett.* **89**, 113509 (2006).

8. Liao, M. Y., Wang, X., Teraji, T., Koizumi, Y. & Koide, Y. Light intensity dependence of photocurrent gain in single crystal diamond photodetectors. *Phys. Rev. B* **81**, 033304 (2010).

9. Sang, L. W., Liao, M. Y., Koide, & Y. Sumiya, M. High-performance metal-semiconductor-metal InGaN photodetectors using CaF_2 as the insulator. *Appl. Phys. Lett.* **98**, 103502 (2011).

10. Arnold, S. P., Prokes, M., Perkins, F. K. & Zaghloul, M. E. Design and performance of a simple, room-temperature Ga_2O_3 nanowire gas sensor. *Appl. Phys. Lett.* **95**, 103102 (2009).

11. Zou, R. J. *et al.* A mobile Sn nanowire inside a beta-Ga_2O_3 tube: a practical nanoscale electrically/thermally driven switch. *Small* **7**, 3377-3384 (2011).

12. Li, L. *et al.* Single-crystalline CdS nanobelts for excellent field-emitters and ultrahigh quantum-efficiency photodetectors. *Adv. Mater.* **22**, 3161 (2010).

13. Gao, T., Li, Q. H. & Wang, T. H. US nanobelts as photoconductors. *Appl. Phys. Lett.* **86**, 173105 (2005).

14. Lu, M. L., Weng, T. M., Chen, J. Y. & Chen, Y. F. Ultrahigh-gain single SnO_2 nanowire photodetectors made with ferromagnetic nickel electrodes. *NPG Asia Mater.* **4**, e26 (2012).

15. Wu, J. M. & Kuo, C. H. Ultraviolet photodetectors made from SnO_2 nanowires. *Thin Solid Films* **517**, 3870-3873 (2009).

16. Hu, L. F., Yan, J., Liao, M. Y., Wu, L. M. & Fang, X. S. Ultrahigh external quantum efficiency from thin SnO_2 nanowire ultraviolet photodetectors. *Small* **7**, 1012-1017 (2011).

17. Wang, Z. L. Zinc oxide nanostructures: growth, properties and applications. *J. Phys. Condens. Mat.* **16**, 829-858 (2004).

18. Soci, C. *et al.* ZnO nanowire UV photodetectors with high internal gain. *Nano Lett.* **7**, 1003-1009 (2007).
